# Group-based trajectory modeling to describe the geographical distribution of tuberculosis notifications

**DOI:** 10.1186/s12889-025-22083-x

**Published:** 2025-02-27

**Authors:** Alemnew F. Dagnew, Colleen F. Hanrahan, David W. Dowdy, Neil A. Martinson, Limakatso Lebina, Bareng A. S. Nonyane

**Affiliations:** 1 Clinical Development, Gates Medical Research Institute One Kendall Square, Building 600, Suite 6-301, Cambridge, MA USA; 2 Department of Epidemiology, Johns Hopkins Bloomberg School of Public Health, Johns Hopkins University, Baltimore, MD USA; 3 Perinatal HIV Research Unit (PHRU), University of the Witwatersrand, Johannesburg, South Africa; 4 Johns Hopkins University Center for TB Research, Baltimore, MD USA; 5 Department of International Health, Johns Hopkins Bloomberg School of Public Health, Johns Hopkins University, Baltimore, MD USA

**Keywords:** Tuberculosis, Group-based trajectory modeling, Latent class growth analysis, Notification

## Abstract

**Background:**

Tuberculosis (TB) is a major public health problem, and understanding the geographic distribution of the disease is critical in planning and evaluating intervention strategies. This manuscript illustrates the application of Group-Based Trajectory Modeling (GBTM), a statistical method that analyzes the evolution of an outcome over time to identify groups with similar trajectories. Specifically, we apply GBTM to identify the evolution of the number of TB notifications over time across various geographic locations, aiming to identify groups of locations with similar trajectories. Locations sharing the same trajectory may be considered geographic TB clusters, indicating areas with similar TB notifications. We used data abstracted from clinic records in Limpopo province, South Africa, treating the clinics as a proxy for the spatial location of their respective catchment areas.

**Methods:**

Data for this analysis were obtained as part of a cluster-randomized trial involving 56 clinics to evaluate two active TB patient-finding strategies in South Africa. We utilized GBTM to identify groups of clinics with similar trajectories of the number of TB patients.

**Results:**

We identified three trajectory groups: Groups 1, comprising 57.8% of clinics; Group 2, 33.9%; and Group 3, 8.3%. These groups accounted for 30.8%, 44.4%, and 24.8% of total TB-diagnosed patients, respectively. The estimated mean number of TB-diagnosed patients was highest in trajectory group 3 followed by trajectory group 2 across the 12 months, with no overlap in the corresponding 95% confidence intervals. The estimated mean number of TB-diagnosed patients over time was fairly constant for trajectory groups 1 and 2 with exponentiated slopes of 0.979 (95% CI: 0.950, 1.004) and 1.004 (95% CI: 0.977, 1.044), respectively. In contrast, there was a statistically significant 3.8% decrease in the number of TB patients per month for trajectory group 3 with an exponentiated slope of 0.962 (95% CI: 0.901, 0.985) per month.

**Conclusions:**

GBTM is a powerful tool for identifying geographic clusters of varying levels of TB notification when longitudinal data on the number of TB diagnoses are available. This analysis can inform the planning and evaluation of intervention strategies.

**Supplementary Information:**

The online version contains supplementary material available at 10.1186/s12889-025-22083-x.

## Background

Globally, tuberculosis (TB) killed 1.25 million people in 2023, caused almost twice as many deaths as HIV/AIDS, and has probably returned as the leading cause of death from a single infectious agent after COVID-19 has replaced it for 3 years [1]. South Africa is among the countries with the highest annual incidence of TB at 427 patients per 100,000 people in 2023 [1]. The first national TB prevalence survey in South Africa reported a prevalence of 852 bacteriologically confirmed pulmonary TB per 100,000 people among individuals 15 years and older in 2018, and the estimated prevalence of all forms of TB in all ages was 737 per 100,000 people [2].

For resource planning and evaluation of existing or new TB control strategies, reliable data on the burden of TB across different geographical areas within a country or a region is critical. However, it can be challenging to obtain a reliable denominator to determine area-based TB burden (incidence or prevalence) in resource-limited settings [3]. An alternative strategy that uses routinely collected aggregate data at a health facility or other administrative level may help identify high TB notification areas. We consider an analytical method that can be applied to either known incidence /prevalence data or routinely collected health facility notifications over time.

Group-Based Trajectory Modeling (GBTM), also known as Latent Class Growth Analysis (LCGA), is a statistical methodology for analyzing the evolution of an outcome over time [4]. It is a semi-parametric finite mixture model for longitudinal data that assumes data generation is from a finite mixture of distributions, and it identifies groups/classes of individuals with a similar trajectory [5].

This paper illustrates the use of GBTM to describe TB disease trajectories using facility-level data obtained as part of a cluster-randomized trial to evaluate two strategies of active TB case finding in two rural districts of the Limpopo province in South Africa – the Kharitode TB trial [6]. We hypothesized that the GBTM determines trajectory groups by identifying groups of clinics that share similar trajectories of TB notifications over time [5, 7, 8]. This analysis uses facilities/clinics as a proxy for spatial location and the clinic-abstracted longitudinal data as a proxy for notification of the disease in the facilities' catchment areas.

## Methods

### Trial design and setting

The Kharitode TB trial was a cluster-randomized, open-label, parallel-controlled trial to evaluate two active TB patient-finding strategies (facility-based and contact tracing) in Waterberg and Vhembe districts in Limpopo Province, South Africa as described by Hanrahan et al. [6]. Based on willingness to participate, quality of record keeping, and reachable geographic location, the 56 largest public-sector primary care clinics from the two districts were selected. They were randomized in a 1:1 ratio to the facility-based or contact-tracing patient-finding strategies.

### Primary outcome

The primary outcome for our current analysis was the number of people diagnosed with TB and started on treatment in each of the 56 facilities between January and December 2017, which was collected as part of the Kharitode TB trial [6]. Additional covariates available in the trial database were clinic-level TB patient volume (historic TB volume) strata based on the pre-trial period annual number of patients started on treatment and patient-level characteristics [age, sex, HIV (human immunodeficiency virus) status, ART (antiretroviral therapy) status, sputum smear test results].

### Sample size and sampling method

In the Kharitode TB trial, 3,655 TB-diagnosed patients started treatment across 56 facilities between July 2016 and January 2018 [6].

To illustrate trajectory modeling to identify geographic clusters of varying TB notification areas, we analyzed TB treatment initiations reported in the calendar year of 2017 thus capturing time trends over the calendar year.

### Data management

For the trajectory modeling, we calculated clinic-level total counts of TB treatment initiations each month. Percentages for clinic-level characteristics (intervention arm, historic TB volume strata, and district) and clinic-level summaries (means, medians, standard deviations, and percentages as appropriate) of the patient-level characteristics were generated. Trends of the TB-treatment initiations over time are illustrated using line graphs*.*

### Group-Based Trajectory Modeling (GBTM)

We used the TRAJ package in STATA 17.0 for our analyses [9]. Considering the outcome was a count with notable extra-variability and zero values, we applied GBTMs assuming a Zero Inflated Poisson (ZIP) distribution. We evaluated a fit of various functional forms for the relationship between the outcome and time, including linear, quadratic, and cubic trajectory functions, and we selected the model with the lowest Bayesian information criterion (BIC) and AIC (Akaike information criterion), and the percentage of clinics in each trajectory group greater than 5% as recommended by Nguena Nguefack et al*.* [5, 10]. To ensure parsimony, models with statistically non‐significant (*p*-value > 0.01) cubic and quadratic parameters (intercepts and slopes) were not selected even if they fulfilled the other selection criteria [10, 11].

To further evaluate the accuracy of the selected model, we calculated the average posterior probability (AvePP) for each trajectory group by assigning the maximum of the three posterior trajectory probabilities of each clinic to their trajectory group and adding those probabilities, and dividing by the number of clinics in their respective trajectory groups [5, 10, 12, 13]. In addition, we calculated the odds of correct classification (OCC) for each trajectory group by dividing the odds obtained from the AvePP (Observed) by the odds obtained from the probabilities/percentages of clinics assigned to each trajectory group (expected). The AvePP was used to measure the certainty of assigning clinics to their most likely group, while the OCC quantified the model's ability to discriminate between groups by comparing the odds of correctly classifying clinics to the odds of misclassifying them. The OCC incorporates both the AvePP and the expected odds based on group size. In the OCC calculation, the expected odds serve as the denominator, adjusting for group size to prevent large groups from artificially inflating OCC values. We then assessed the accuracy of the selected model based on AvePP > 0.70 and OCC > 5.0 [5, 10, 12, 13].

Bootstrapping with 1000 repeats was used to calculate the 95% CI for the GBTM parameters (intercepts and slopes). We exponentiated the estimates and the corresponding 95% CIs to get the final results.

### Association between the GBTM trajectory groups and clinic-level characteristics

We performed Fisher's exact test to assess the association between the trajectory groups and clinic-level variables: intervention arm (from the Kharitode TB trial), historic TB volume strata, and district. To evaluate the association between the trajectory groups (regrouped trajectory groups 3 and 2 into one stratum as there were only five clinics in trajectory group 3) and the clinic-level variables (regrouped high and medium historic TB volume strata into one stratum as there were only two clinics in the high stratum), we estimated the unadjusted risk ratios and 95% confidence intervals using log-binomial regression models. For the adjusted risk ratios and 95% confidence intervals, we used the multivariable Poisson regression model with robust variance (an approximation to log-binomial regression models).

## Results

### Clinic and patient characteristics

Among all 3655 TB patients included in the Kharitode trial, 2141 started TB treatment from January through December 2017 and were included in the analyses reported here. Their median age was 38 years; 59% were male, and 63% were living with HIV (Table 1). The characteristics of the Kharitode trial clinics and patients were similar between the Kharitode trial arms (Supplemental Table 1).

**Table 1:** Characteristics of the Kharitode trial clinics and patients who started tuberculosis treatment in 2017 in Limpopo Province, South Africa

Characteristic	Overall (*N* = 2141)
Median age (year)	38.0
Male sex – No. (%)	1254 (59)
Smear positive – No. (%)	523 (24)
Living with HIV – No. (%)	1341 (63)
On ART – No. (%)^a^	1204 (90)
Clinics by district – No. (%)	
Waterberg	28 (50)
Vhembe	28 (50)
Clinics by historic TB volume –No. (%)	
Low	40 (71)
Medium	14 (25)
High	2 (4)

N = total number of patients; No. (%) = number (percentage) of patients/clinics in each category; *HIV* Human immunodeficiency virus, *ART* Antiretroviral therapy

^a^The percentage is among HIV positives

The monthly number of TB patients who started treatment in the 56 clinics ranged from 0 to 19; most were below 10, and 13% of these monthly reports were zero values.

### Group-Based Trajectory Modeling (GBTM) results

Among all the fitted GBTMs, a model with three trajectory groups and linear functions that fulfilled the selection criteria was selected. The three trajectory groups' AvePP (membership probability) and OCC were higher than 0.70 and 5, respectively (Table 2).

**Table 2:** Model accuracy

Trajectory group	AvePP	OCC
Group 1	1.00	211
Group 2	0.97	73
Group 3	0.93	138

*AvePP *Average posterior probability, *OCC* Odds of correct classification

High AvePP values greater than 0.70 indicate strong certainty regarding group membership for clinics within that group, reflecting a well-fit model. High OCC values greater than 5 demonstrate that the model classifies clinics into the correct group with much higher confidence than the likelihood of misclassification, indicating strong group discrimination (effective separation of groups).

**Fig. 1:**
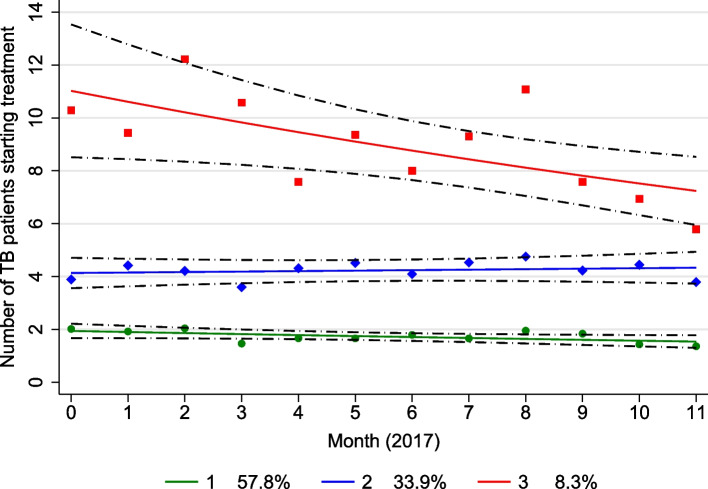
Group-based trajectory modeling (GBTM): Estimated trajectory groups (green, blue, and red solid lines for trajectory groups 1, 2 & 3, respectively), observed group means at each month (green circle, blue diamond and red square symbols for trajectory groups 1, 2 & 3, respectively), and estimated group percentages. The black dash-dot lines are 95% pointwise confidence intervals on the estimated trajectory groups

Based on the selected model, we identified three trajectory groups that included 57.8% (group 1), 33.9 (group 2) and 8.3% (group 3) of the clinics (Fig. 1). These groups accounted for 30.8%, 44.4%, and 24.8% of total TB-diagnosed patients, respectively. The model closely predicted the observed number of TB-diagnosed patients started on treatment and there was more between-month variability for group 3 than the other groups (Fig. 1). The estimated mean number of TB-diagnosed patients was highest at all time points (months) in trajectory groups 3, followed by group 2 and then 1, with no overlap in the 95% confidence intervals between the three trajectory groups (Fig. 1).

**Fig. 2:**
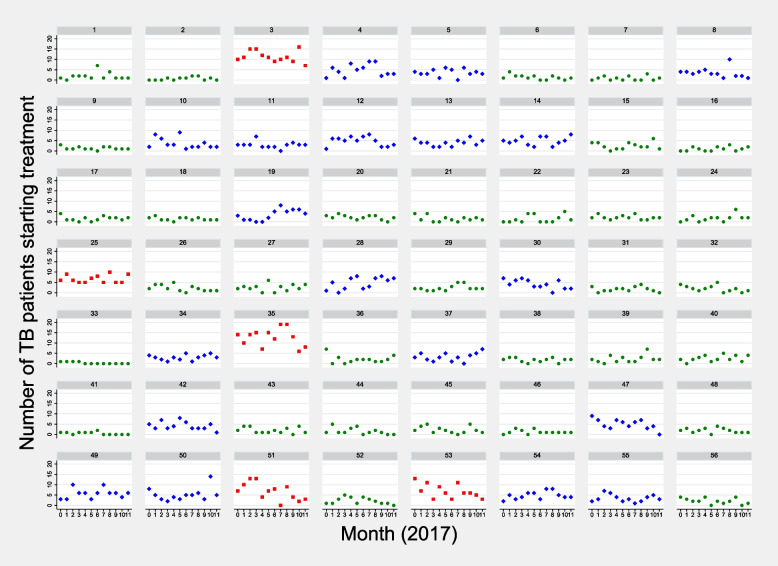
Scatter plot with the month of TB treatment start [Month 0 (January) to Month 11 (December)] in the X-axis and the number of TB patients who started treatment in the Y-axis. The numbers 1 to 56 represent the 56 clinics, and the green circles, blue diamonds, and red squares denote the monthly number of TB patients at each clinic, categorized into trajectory groups 1, 2, and 3, respectively

**Fig. 3:**
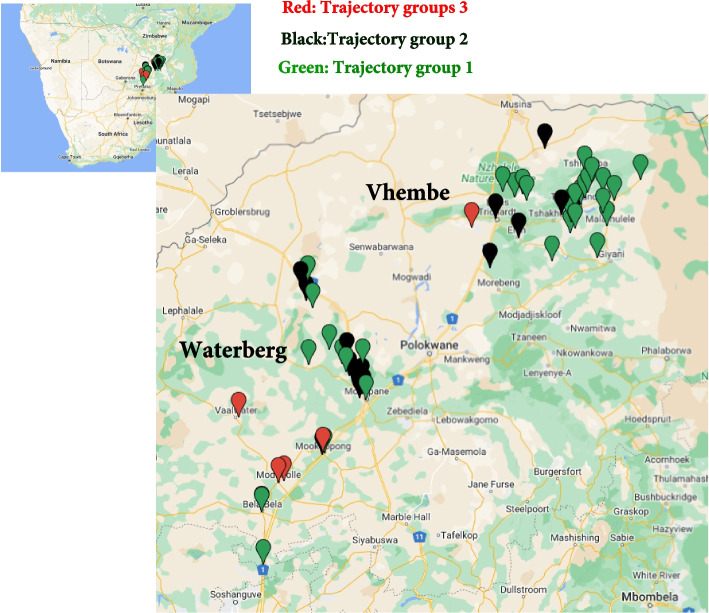
Map showing the spatial distribution of the 56 clinics/facilities: Map markers or map icons represent each of the 56 clinics (green, black, and red for trajectory groups 1, 2 & 3, respectively)

In this analysis, facilities/clinics were used as a proxy for spatial location, and the clinic-abstracted longitudinal data serve as a proxy for the background notification of the disease in the facilities' catchment areas. Therefore, we considered clinics belonging to trajectory groups 3, 2, and 1 as proxies for high, medium, and low geographic clusters of TB notification areas, respectively. The list of clinics in each trajectory group by district is presented in Table 3. The monthly observed number of TB patients in each of the 56 clinics, along with their corresponding trajectory groups, is shown in Fig. 2. We illustrate the spatial distribution of these facilities on a map (Fig. 3), and this clearly shows the four Waterberg facilities with the highest TB notifications in close proximity to each other, the area within this district that is dominated by Group 2 facilities, as well as the north-eastern corner of the Vhembe district which is dominated by low notification facilities belonging to Group 1.

**Table 3:** List of the 56 clinics (dummy clinic numbers) by trajectory groups and district

District	Group 1 (*N* = 32)	Group 2 (*N* = 19)	Group 3 (*N* = 5)
**Waterberg**	**Clinic numbers** **(*n* = 11)**	**Clinic numbers** **(*n* = 13)**	**Clinic numbers** **(*n* = 4)**
	1, 2, 17, 20, 26, 27, 29, 33, 38, 40, 41	4, 8, 13, 14, 19, 28, 30, 34, 37, 42, 49, 54, 55	3, 25, 35, 53
**Vhembe**	**Clinic numbers** **(*n* = 21)**	**Clinic numbers** **(*n* = 6)**	**Clinic numbers** **(*n* = 1)**
	6, 7, 9, 15, 16, 18, 21, 22, 23, 24, 31, 32, 36, 39, 43, 44, 45, 46, 48, 52, 56	5, 10, 11, 12, 47, 50	51

N = number of clinics in each trajectory group; n = number of clinics in a district within each trajectory group

The intercepts (estimated mean number of TB patients) and their corresponding 95% CI for trajectory groups after centering at Month 5 (June) are shown in Table 4. For trajectory groups 1 and 2, the estimated mean number of TB patients over time remained constant with exponentiated slopes of 0.979 (95% CI: 0.950, 1.004) and 1.004 (95% CI: 0.977, 1.0439), respectively (Table 4). However, there was a statistically significant 3.8% decrease in the estimated mean number of TB patients per month for trajectory group 3 [exponentiated slope of 0.962 95% CI: 0.901, 0.985)] (Table 4).

**Table 4:** Intercepts and slopes for three trajectory groups estimated using Group-based trajectory modeling

Trajectory	Intercept^a^	Slope
	**Estimate (95% CI)**	**Estimate (95% CI)**
**Group 1**	1.748 (1.455, 1.990)	0.979 (0.950, 1.004)
**Group 2**	4.224 (3.383, 5.052)	1.004 (0.977, 1.0439)
**Group 3**	9.105 (5.822, 12.747)	0.962 (0.901, 0.985)

^a^Intercept after centering at Month 5 (June), *CI* Confidence Interval

### Results of the association between the GBTM trajectory groups and clinic-level characteristics

As shown in Table 5, Fisher's exact test revealed no statistically significant association between trajectory group and trial arm (*p*-value = 0.99). However, significant associations were observed between trajectory group and district (*p*-value = 0.026), as well as trajectory group and historic TB volume strata (*p*-value < 0.001).

**Table 5:** Number of clinics in each of the trajectory groups by clinic characteristics

Characteristic	Group 1 (*N* = 32)	Group 2 (*N* = 19)	Group 3 (*N* = 5)	*P*-value*
**Trial arm – No. (%)**				
Contact-tracing	16 (50.0)	9 (47.4)	3 (60.0)	1.00
Facility-based	16 (50.0)	10 (52.6)	2 (40.0)	
**District – No. (%)**				
Waterberg	11 (34.4)	13 (68.4)	4 (80.0)	0.026
Vhembe	21 (65.6)	6 (31.6)	1 (20.0)	
**Historic TB volume – No. (%)**				
Low	30 (93.8)	10 (52.6)	0 (0.0)	< 0.001
Medium	2 (6.2)	9 (47.4)	3 (60.0)	
High	0 (0.0)	0 (0.0)	2 (40.0)	

N = total number of clinics in a trajectory group; No. = number of clinics in each category

^*^Fisher's exact test

To evaluate the association between the GBTM trajectory groups and clinic-level characteristics, we performed univariate and multivariate analyses (Supplemental Table 2). In univariate analysis, clinics in Waterberg district were 2.4 (95% CI: 1.2, 4.9) times more likely to belong to trajectory group 3 (high geographic cluster of TB notification area) or trajectory group 2 (medium geographic cluster of TB notification area) than clinics in Vhembe district; this relative risk was 2.0 (95% CI: 1.1, 3.9) after adjusting for trial arm and historic TB volume strata. In univariate analysis, clinics in the high or medium stratum of historic TB volume strata were 3.5 (95% CI: 2.0, 6.2) times more likely to belong to the high or medium geographic clusters of TB notification areas than those in the low stratum historic TB volume; this relative risk was 3.1 (95% CI: 1.8, 5.6) after adjusting for the trial arm and district. The median age of patients in the three trajectory groups during the months of TB treatment [Month 0 (January) to Month 11 (December)] ranged from 34 to 44 years. No consistent patterns were observed in the median age or other patient characteristics across the trajectory groups or treatment months (Supplemental Table 3).

## Discussion

Using the number of people who started treatment for TB in the calendar year 2017 among 56 South African clinics, we have illustrated the use of GBTMs to identify geographical clusters of varying TB notifications. The GBTM model with three trajectory groups and linear function was found to be the most suitable and the selected model that fulfilled the recommended criteria, such as having the lowest BIC and AIC and the percentage of clinics in each trajectory group greater than 5% [5, 10–13]. In addition, each of the three trajectory groups had high AvePP (strong certainty regarding group membership for clinics within a group) and OCC (effective separation of groups), indicating the accuracy of the model [5, 10, 12, 13].

The estimated mean number of TB patients in the three trajectory groups differed from each other at all time points during the 12 months, with no overlap in their 95% confidence intervals. Using clinics/facilities as a proxy for spatial location and the clinic-abstracted longitudinal data as a proxy for background notification of the disease in the clinic's catchment areas, we considered clinics belonging to trajectory groups 3, 2, and 1 as high, medium, and low TB notification areas, respectively [13].

The estimated mean number of TB diagnoses over the 12 months in 2017 was constant in trajectory groups 1 and 2, while there was a statistically significant 3.8% decrease per month in the estimated mean number of TB patients per month for trajectory group 3. In addition, our analysis indicated that contemporaneous trajectories were positively associated with past reporting of TB diagnoses, as all clinics with high or medium historic TB volume were assigned to trajectory group 3 by our analyses.

There was no association between Kharitode TB trial arm and the trajectory group, emphasizing that the two TB patient-finding strategies (facility-based and contact tracing) resulted in similar monthly notifications over time in line with the primary analysis of Kharitode TB trial of overall notifications and TB treatment initiations during the trial period [6]. Facilities/Clinics from the Waterberg district, which is peri-urban, were more likely to belong to high or medium geographic clusters of TB notification areas compared to the more rural Vhembe facilities. Prior data from South Africa suggests more densely populated urban areas have a high burden and risk of TB [14, 15].

We observed a decline in TB diagnoses specifically in the high-notification group (group 3). While this may reflect regression to the mean over time, it is also possible that declines in TB incidence and notifications—which have been observed nationwide in South Africa over the past decade [1]—are concentrated in areas with high existing TB incidence. This result can be useful to decision-makers as they seek to effectively allocate resources for TB control in these clinics and in South Africa more broadly. Specifically, it may be that additional resources are best focused on high-notification clinics where existing incidence is highest and opportunity for meaningful progress is greatest.

The median age, proportion of males, proportion of people living with HIV, and proportion of people on ART included in this report were similar to the Kharitode TB trial by trial arm and overall [6]. In addition, the median age, the proportion of males, and the proportion of HIV positives were similar to the national data [2].

We note that this method is a descriptive tool that classifies geographical locations according to varying levels of the outcome of interest. It does not attempt to identify risk factors associated with the outcome, but as illustrated, additional analyses can be conducted, for example, for the purpose of evaluating the association between trajectory groups and the population characteristics of interest, such as population mobility, population density, HIV prevalence, etc.

The GBTM was applied to aggregate longitudinal data to identify TB geographic clusters of high or low TB notification areas. In our scenario, where the denominator population is unknown, we made the assumption that notifications are a proxy for the true burden in the catchment areas [3]. This GBTM analysis identified groups of clinics with similar trajectories of TB patient numbers, which can aid in resource planning and evaluating intervention strategies.

One limitation of this analysis is its assumption that clinics within the same trajectory groups will follow the same trajectories, potentially overlooking variations in individual clinic trajectories. However, the primary focus is on the grouping based on existing data, rather than the precise shape of the curve. Our model was shown to be highly accurate, as indicated by the AvePP and OCC metrics.

## Conclusion

GBTM is a powerful tool for identifying geographic clusters with varying levels of TB notifications. We demonstrated this using facility-abstracted TB patient counts as a proxy for TB notifications in the facilities' catchment areas. This analytical approach can inform the planning and evaluation of intervention strategies for TB and other communicable diseases.

## Supplementary Information

Supplementary Material 1.

## Data Availability

Kharitode trial data are available in summary within the primary manuscript and all other relevant data belong to the South Africa National Department of Health. Restrictions apply to the availability of these data which were used under license for this study. Data are available with the permission of https://www.health.gov.za/.

## Abbreviations

AIC: Akaike information criterion
AvePP: Average posterior probability
BIC: Bayesian information criterion
ART: Antiretroviral therapy
GBTM: Group-based trajectory modeling
HIV: Human immunodeficiency virus
LCGA: Latent class growth analysis
OCC: Odds of correct classification (OCC)
TB: Tuberculosis
WHO: World Health Organization
ZIP: Zero inflated poisson
