# Supplementary material for: Group-based trajectory modeling to describe the geographical distribution of tuberculosis notifications
Source: BMC Public Health. 2025 Feb 27;25:797. doi: 10.1186/s12889-025-22083-x (PMC11866561; doi:10.1186/s12889-025-22083-x)
Supplement: Supplementary file 1 — Supplementary Material 1. [file 12889_2025_22083_MOESM1_ESM.docx]

**Supplementary Table 1. Characteristics of the trial patients who started tuberculosis treatment in 2017 in Limpopo Province, South Africa**

| **Characteristic** | **Facility-based screening arm**  **(N= 1144)** | **Contact-tracing arm (N= 997)** | **Overall**  **(N= 2141)** |
| --- | --- | --- | --- |
| Median age (year) | 39.0 | 38.0 | 38.0 |
| Male sex – No. (%) | 681 (59) | 573 (57) | 1254 (59) |
| Smear positive – No. (%) | 259 (23) | 264 (26) | 523 (24) |
| HIV positive – No. (%) | 715 (62) | 626 (63) | 1341 (63) |
| On ART – No. (%)* | 643 (90) | 561 (90) | 1204 (90) |
| Clinics by district – No. (%)  Waterberg  Vhembe | 14 (50)  14 (50) | 14 (50)  14 (50) | 28 (50)  28 (50) |
| Clinics by historic TB volume –No. (%)  Low  Medium  High | 20 (71)  7 (25)  1 (4) | 20 (71)  7 (25)  1 (4) | 40 (71)  14 (25)  2 (4) |

N = total number of patients; No. (%) = number (percentage) of patients/clinics in each category; HIV = Human immunodeficiency virus; ART = antiretroviral therapy; *= The percentage is among HIV positives

**Supplementary Table 2. Association of Clinic-Level Characteristics with Membership in Trajectory Group 3 or Group 2 Compared to Group 1**

| **Characteristic** | **Univariate analysis** | | **Multivariate analysis** | |  |
| --- | --- | --- | --- | --- | --- |
|  | | **Unadjusted RR (95% CI)** | | **Adjusted RR (95% CI)** | |
| **Trial arm** (Contact-tracing vs Facility-based) | | 1 (0.5, 1.8) | | 0.9 (0.6, 1.5) | |
| **District** (Waterberg vs Vhembe) | | 2.4 (1.2, 4.9) | | 2.0 (1.1, 3.9) | |
| **Historic TB volume** (High or Medium vs Low) | | 3.5 (2.0, 6.2) | | 3.1 (1.8, 5.6) | |

RR = Relative Risk; CI = Confidence Interval

**Supplementary Table 3. Characteristics of the trial patients who started tuberculosis treatment in 2017 in Limpopo Province by trajectory group and time, South Africa**

| **Characteristic** | **M0**  **(N= 187)** | **M1**  **(N= 190)** | **M2**  **(N= 203)** | **M3**  **(N= 165)** | **M4**  **(N= 171)** | **M5**  **(N= 183)** | **M6**  **(N= 173)** | **M7**  **(N= 183)** | **M8**  **(N= 205)** | **M9**  **(N= 175)** | **M10**  **(N= 163)** | **M11**  **(N= 143)** |
| --- | --- | --- | --- | --- | --- | --- | --- | --- | --- | --- | --- | --- |
| Median age (year)  Group 1  Group 2  Group 3 | 38  37  38 | 39  41  39 | 38  41  36 | 40  40  34 | 37  38  40 | 36  36  38 | 44  38  38 | 36  40  35 | 41  39  44 | 39  37  37 | 42  39  36 | 35  37  38 |
| Male sex (%)  Group 1  Group 2  Group 3 | 66  56  64 | 52  65  55 | 60  53  61 | 60  67  69 | 64  62  51 | 74  54  63 | 49  59  55 | 52  65  44 | 55  58  51 | 53  64  54 | 71  58  56 | 51  51  53 |
| Smear positive (%)  Group 1  Group 2  Group 3 | 50  32  28 | 31  17  21 | 25  25  20 | 33  29  20 | 25  12  19 | 38  29  30 | 23  16  18 | 33  17  20 | 29  22  27 | 36  23  22 | 18  13  39 | 19  22  10 |
| HIV positive (%)  Group 1  Group 2  Group 3 | 55  62  66 | 64  57  68 | 57  70  71 | 62  65  65 | 51  68  76 | 53  64  70 | 68  70  78 | 72  69  71 | 56  66  55 | 43  59  62 | 44  64  50 | 63  64  47 |
| On ART (%)*  Group 1  Group 2  Group 3 | 86  91  91 | 90  87  88 | 86  96  98 | 86  100  94 | 100  96  93 | 82  91  100 | 92  87  97 | 92  83  91 | 86  81  83 | 88  94  87 | 90  78  88 | 96  86  79 |

M = month; N = total number of patients; % = percentage of patients in each category; HIV = Human immunodeficiency virus; ART = antiretroviral therapy; *= The percentage is among HIV positives
